# Metabolic Footprint of *Drosophila* S2 Cells: Findings During the Production of a Recombinant Rabies Virus Glycoprotein

**DOI:** 10.1002/biot.70307

**Published:** 2026-09-10

**Authors:** Monize Caiado Decarli, Diogo Peres dos Santos, Daniela Matilde Correia, Amadeus Gomes de Azevedo, Claudio Alberto Torres Suazo, Ângela Maria Moraes, Renato Mancini Astray

**Affiliations:** ^1^ Department of Chemical Engineering Federal University of São Carlos São Carlos São Paulo Brazil; ^2^ Department of Engineering of Materials and of Bioprocesses School of Chemical Engineering University of Campinas São Paulo Brazil; ^3^ Department of Biomaterials & Biomedical Technology University Medical Center Groningen/University of Groningen Groningen the Netherlands; ^4^ National Institute for Bioprocessing Research and Training (NIBRT) Dublin Ireland; ^5^ Multipurpose Laboratory Butantan Institute São Paulo Brazil

**Keywords:** cell metabolism, *Drosophila;* rabies vaccine, metabolic footprint, S2 cells, vaccine production

## Abstract

Over the past 50 years, the *Drosophila melanogaster* S2 cells have been valued for their ability to synthesize therapeutic molecules at high yield. To further increase protein expression, it is imperative to improve cellular performance, which is intrinsically linked to cell metabolism. Nevertheless, information on S2 metabolism, including pathways, components, and cellular compartments, remains limited, hindering advances in S2 cellular performance. Herein, using a genetically modified S2 cell line expressing the recombinant rabies virus glycoprotein (RVGP), we investigated the stress caused by RVGP production on S2 cells. Batch cultures using wild‐ and rec‐types were performed, and 27 compounds were quantified over 192 h. The extracellular metabolome affected the rec‐S2 growth kinetics after RVGP expression was activated. Although RVGP was produced in high amounts, we identified a substrate limitation for rec‐S2 cell growth (glutamine), changes in amino acid routes due to RVGP biosynthesis (leucine, serine, glycine, and valine), and metabolites that might be affecting rec‐S2 cell growth (acetate, pyruvate, citrate, and malate). Organic acid analysis indicated that malate and acetate production are correlated with RVGP production. This work revealed metabolic correlations in S2 cells that may have direct implications for media optimization and yield maximization, thereby improving S2 performance for scale‐up.

## Graphical Abstract and Lay Summary

To further increase protein expression, it is imperative to improve cellular performance, a process intrinsically linked to cell metabolism. Hence, a metabolic footprint of *Drosophila* S2 cells, both recombinant‐ and wild‐types, was performed to enhance the production of the recombinant rabies virus glycoprotein.

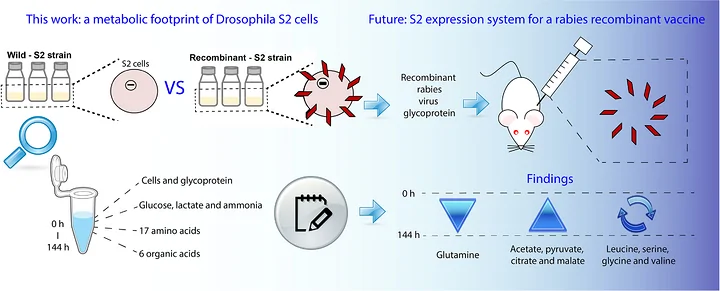

## 1 Introduction

The fruit fly *Drosophila melanogaster (D. melanogaster)* has been one of the most studied organisms in biology due to its simple genome, short life cycle of about 2 weeks, and easy culture conditions. Over the last century, *D. melanogaster* has played a starring role as a genetic model for revealing fundamental biological principles and processes, ranging from innate immunity and inflammation to disease processes such as cancer [1, 2]. Despite extensive study, the metabolic behavior of *D. melanogaster* under stressful conditions has been rarely reported.

In the past 50 years, a cell line derived from *D. melanogaster* named Schneider 2 or S2 [3] has been extensively used for the recombinant production of therapeutic proteins, especially for the investigation of potential candidates for vaccine production [4, 5, 6]. This is due to the advantages of using S2 cells, which are very robust and grow rapidly in serum‐free media, either as loose monolayers or in suspension, without the need for microcarriers. S2 cells also do not require a strictly controlled CO_2_ atmosphere or temperature as high as those required by mammalian cells, growing well at around 28°C [5, 7]. As reported by our team, S2 cells have shown potential for expressing high levels of proteins [5], as well as for enabling the production of complex molecules (e.g., transmembrane and glycosylated) or difficult‐to‐express proteins (e.g., unstable at a certain pH, followed by easily unfolding) [4, 8, 9]. Some examples of candidates for vaccine production that were successfully produced by S2 cells are the reticulocyte‐binding protein, a candidate for the malaria vaccine [10], the Ebola virus‐like particles [11], the truncated dengue virus envelope [12] and the HIV‐1 virus‐like particle [13].

The S2 cell line has also been evaluated for the production of the rabies virus glycoprotein (RVGP) [5, 14]. RVGP is the main antigen of the rabies virus and can induce virus‐neutralizing antibodies that confer protection against rabies virus infection [15]. RVGP is known as a difficult‐to‐express protein due to its hydrophobicity and low stability at pH 6. Besides that, it needs to be covalently attached to a carbohydrate (glycosylation) for proper folding to enable its function [16]. S2 cells do not exhibit the same protein glycosylation capabilities as mammalian cells [7]. However, unlike other insect or microbial systems, the S2 glycosylation profile meets the specific requirements for RVGP production [17]. In fact, RVGP has already been produced in mammalian cells, and the protein quality was similar to that derived from S2 cells, depending on culture conditions, host factors, and glycosylation patterns [5]. Given that the production process of therapeutic proteins using the S2 cells is significantly less complex than procedures involving mammalian cells, requiring less infrastructure and lower overall costs, S2 cells are particularly attractive for this purpose.

To control RVGP expression and function, the S2 cell line was engineered to respond to metal ions supplemented in the culture medium for further scale‐up in a classical stirred‐vessel bioreactor [18]. In this way, the S2 cell machinery is induced to produce RVGP upon the addition of copper sulfate, which occurs only when the bioreactor culture conditions are optimal, thereby avoiding conditions that promote RVGP instability and degradation. The initial steps toward scaling up RVGP production using the inducible metallothionein (S2‐RVGP‐Mt) promoter were successfully demonstrated by our team with bioreactors in two configurations [17, 18]. First, using a classic stirred vessel with a pitched blade impeller, 3 mg/L of RVGP was obtained [18]. Later, this cell line was modified to add a histidine tag for purification (S2‐RVGP‐Mt‐His). This newly developed cell line was cultured in a single‐use bioreactor to reduce the need for cleaning and sterilization. Additionally, this bioreactor employed a wave‐induced agitation system rather than mechanical impellers, which is advantageous in reducing shear stresses during large‐scale culture. Using this single‐use wave bioreactor, 1 mg/L RVGP was obtained, with high immunogenic capacity assessed via an in vivo immunization study [17]. The titer needed for immunization studies can vary depending on the animal model and experimental design. For our immunization studies, 1 mg of RVGP per liter per batch would be sufficient to immunize 500 mice. Hence, both studies [17, 18] demonstrated potential for industrial scale‐up.

To obtain higher titers of recombinant proteins compatible with industrial levels, it is imperative to improve cellular performance [19]. Extensive characterization related to S2 culture parameters and metabolic aspects has been published elsewhere [5, 14, 18, 20]. However, not in a robust cell line with high expression in suspension culture and high purification efficiency. Moreover, instead of investigating the most common metabolic features in an isolated manner, a more efficient tool is to evaluate cellular performance across a vast repertoire of a wide range of extracellular metabolites by using a metabolomic footprint approach [21, 22, 23]. In this approach, medium culture over the course of cell growth and protein production is collected to identify and quantify the repertoire of extracellular molecules that are consumed as substrates or are secreted as byproducts [24, 25]. In *Drosophila* cells, metabolomic analysis has been used for a wide range of studies focusing on the wild‐type strain [26], but there is no report of a metabolomic footprint analysis comparing wild‐ and recombinant‐type strains carrying an inducible promoter for recombinant protein production.

The expression of foreign proteins is often associated with stress reactions in the host cell because resources are reallocated from host‐cellular processes to heterologous protein production [27, 28]. Hence, this study aims to investigate the stress caused by RVGP production in S2 cells through metabolic footprint analysis of the recombinant‐type strain during RVGP production, compared with the wild‐type strain. To do that, three independent batch cultures were performed, and 27 compounds were identified and quantified for 192 h. We hypothesized that this metabolic footprint analysis would enable further development of upstream engineering approaches, such as media culture optimization and yield maximization, to improve S2 cell performance for scale‐up.

## 2 Materials and Methods

### 2.1 Establishing *Drosophila melanogaster* S2, Wild‐ and Recombinant‐Type Strains

The wild‐type strain of *Drosophila melanogaster* Schneider S2 cells was purchased (DES system, Invitrogen, USA) and adapted to the serum‐free medium SF900 III (ThermoFisher, USA). The recombinant‐type strain was obtained from the wild‐type strain as previously described [18]. Briefly, the rec‐type strain was obtained by the transfection of a plasmid containing both the expression and the selection elements. The RVGP cDNA was cloned downstream of the *Drosophila* inducible metallothionein promoter (Mt) and hygromycin B (H) was used to select the recombinant population. Aiming to facilitate the purification steps, a polyhistidine tag (His‐tag) was also introduced at the C‐terminus of RVGP, as previously described [17]. Due to all these modifications, the resulting recombinant cell line was named S2MtRVGP‐H‐His and is referred to here by the acronym “rec‐S2”, while the wild‐type is referred to as “wild‐S2”.

### 2.2 Culture of *Drosophila melanogaster* S2, Wild‐ and Recombinant‐Type Strains

S2 cell populations are indefinite, and single cells were cultured under static or stirred conditions. In static cultures, wild‐ and rec‐type strains were inoculated at 5 × 10^5^ cells/mL into T‐25 cm^2^ flasks in SF900‐III serum‐free medium (ThermoFisher, USA) at 28°C under normal atmospheric conditions. The rec‐S2 population was established after three passages in a culture medium containing 50 mg.mL^−1^ hygromycin B (Gibco, Germany). Following the same culturing procedure as for the static conditions, 5 × 10^5^ cells/mL of wild‐S2 and rec‐S2 cells were expanded in a working volume of 20 mL of Sf‐900 III culture medium in a 100 mL culture vessel (Schott flask). The Schott flasks were maintained in a rotary incubator (G‐25KC model, New Brunswick Scientific, USA) at 100 rpm for 192 h. To induce RVGP expression in rec‐S2 cells, copper ions (Cu^2+^) were added in a single step at 72 h, in the form of 75 µL of a 0.7 mM CuSO_4_ (Sigma–Aldrich, USA) aqueous solution, in the working volume of 20 mL mentioned above, as previously established [17]. Three independent batch cultures were performed for each strain, each initiated from three independent cryotubes. An additional experiment was performed with the wild‐S2 cells in the presence of CuSO_4_ to rule out potential effects of this compound on these cells.

### 2.3 Sample Harvesting and Preparation for Analysis

Aliquots of 2 mL were collected under mixing conditions and in sterile environments every 48 h for 192 h (days 2, 4, 6, and 8). The pH was immediately measured. On the other days (days 1, 3, 5, and 7), 1 mL aliquots were collected. These aliquots were used for determining cell concentrations every 24 h. Then, aliquots were centrifuged at 600 g for 5 min, and the cell pellet was used for RVGP quantifications. The supernatant was used to analyze 26 compounds, such as glucose, ammonia, 17 amino acids, and 6 organic acids, as described below. No addition of fresh culture medium was performed, otherwise, the reliability of the metabolic footprint in a batch mode would be compromised. To ensure proper aeration, the volume of the culture medium corresponded to 20% of the Schott flask capacity (20 mL in a 100 mL flask), and the flasks were opened for sampling every day under sterile conditions.

### 2.4 Cell Concentration and Viability

Cell concentration and viability were analyzed using a hemacytometer, employing the 0.04% trypan blue (Life Technologies, USA) exclusion method. Two technical replicates (*n* = 2) were collected independently from each Schott flask batch culture (*n* = 3), for a total of *n* = 6.

### 2.5 Recombinant RVGP

For RVGP quantification, 10^6 ^cells were collected and analyzed using an ELISA kit purchased from Pasteur Institute through MTA signed in 2019 (Rabies Glycoprotein Enzyme Immunoassay, Pasteur Institute, France). ELISA analysis was performed checking for standard curve fit based in historical data and quality of reagents following the protocol previously described [17]. The amino acid composition of the RVGP was obtained from the nucleotide sequence (GenBank accession code M13215.1) of the *Pasteur* rabies virus strain.

### 2.6 Glucose, Lactate, and Ammonia

Glucose (Gluc) and lactate (Lac) quantifications were performed in solution by enzymatic analysis using a YSI 7199‐06A bioanalytical system (YSI, Yellow Springs, USA). For ammonia (Amm) quantification, samples were previously adjusted to ionic strength (ISA, Hexis Científica, BRA), and analyzed by a selective ion electrode (Orion 710A, Thermo Scientific, United States). For both analyses, the supernatant was isolated with a previous centrifugation step (600 g for 5 min).

### 2.7 Amino and Organic Acids

Aliquots used for amino and organic acid analysis were previously lyophilized (Freezone 6 Freeze Dry System, Labconco, United States). After lyophilization, amino acids were quantified by high‐performance liquid chromatography (HPLC), using a UV W486 detector at 254 nm, Pico‐tag (300 × 3.9 mm) amino acid analysis column (Waters Corporation, United States), and amino acid standards as reference samples (AccQ‐Tag, Waters Corporation, MA), following a previously described methodology [17]. The mobile phase consisted of two eluents. The first eluent was composed of 6% acetonitrile, 0.14 M sodium acetate trihydrate, 0.05% trimethylamine, and 0.02% ethylenediaminetetraacetic acid (EDTA) in ultrapure water solution. The second eluent was composed of 60% acetonitrile and 0.02% EDTA in ultrapure water. A flow rate of 1 mL/min was utilized, and a gradient elution was used by increasing the amount of the second solution. The amino acids quantified were: alanine (Ala), asparagine (Asn), aspartic acid (Asp), cysteine (Cys), glutamic acid (Glu), glutamine (Gln), glycine (Gly), histidine (His), isoleucine (Ile), leucine (Leu), lysine (Lys), methionine (Met), phenylalanine (Phe), proline (Pro), serine (Ser), tyrosine (Tyr), and valine (Val). Organic acids were also analyzed using the same HPLC equipment mentioned above, with a UV W486 detector at 210 nm, and an Aminex HPX‐87H column (Bio‐Rad, United State**s**), 300 × 3.9 mm, at 65°C. The mobile phase in this case consisted of 0.005 M sulfuric acid solution at a flow rate of 0.6 mL/min. The organic acids quantified were: acetate (Ac), citrate (Cit), lactate (Lac), malate (Mal), propionate (Prop), and pyruvate (Pyr).

### 2.8 Calculation of Parameters Related to Cell Growth and Metabolic Rates

Equation (1) was used to determine the specific growth rate (µ):

(1) $$\mu =\frac{1}{X}\;\frac{dX}{dt}$$

where *X* is cell concentration (cell/mL) and *t* is the time of culture (hours). In the exponential growth phase, the maximum specific growth rate was determined from the linear correlation between the *µ* values obtained. Since it is an inducible expression system and the addition of CuSO_4_ impacts cell growth rates, the specific growth rate before and after induction are represented by *µ*_before_ (0 – 72 h) and *µ*_after_ (72 – 120 h), respectively, instead of a single *µ*_maxim_ that would not be representative as a whole.

To determine the doubling time (d_t_), Equation (2) was used:

(2) $$d_{t}=\frac{Ln\;\left(2\right)}{\mu }$$

To determine the specific consumption of substrates from the extracellular environment, specific substrate uptakes (*q*_s_) were calculated using Equation (3):

(3) $$q_{S_{i}}=-\frac{1}{X}\frac{dS_{i}}{dt}$$

where *i* is a given substrate and *S*_i_ is the concentration of a substrate in mmol/L.

To determine the specific byproduct/metabolite released in the extracellular environment, specific production rates (*q*_p_) were calculated using Equation (4):

(4) $$q_{P_{i}}=\frac{1}{X}\;\frac{dP_{i}}{dt}$$

where *i* is a given byproduct/metabolite and *P_i_* is the concentration in mmol/L for all metabolites/byproducts, except the main product RVGP, which is in ng/mL.

### 2.9 Data Processing for Applying Metabolomics Analysis

The results attained for concentrations of all amino and organic acids by HPLC analysis were thereafter transformed into *Z‐scores*, following the procedure developed elsewhere [22]. First, the values corresponding to the composition of the basal culture medium (day 0, fresh culture medium before starting the culture) were subtracted from the raw data of each replicate. Then, the values were normalized to cell concentration, that is, all values were divided by the corresponding amounts of viable cells present in the culture at each time point. Finally, the mean value was calculated using the three replicates for both wild‐ and rec‐type strains, and these final values were transformed into Z‐scores according to Equation (5):

(5) $$Z\;score_{k,i}=\frac{\left(I_{k,i}-mean\;I_{k,1\text{…}k,n}\right)}{SD_{k,1\text{…}k,n}}$$

where *k* corresponds to each metabolite analyzed and *i* corresponds to a specific time point in the culture. Thus, *I_k_,i* corresponds to a value of a specific metabolite at a defined time point. The mean was calculated for the time points ranging from 0 to 144 h of batch cultures due to the reduction observed in cell viability after this point. *SD* is the standard deviation of the values corresponding to that specific mean.

### 2.10 Statistical Analysis

All experiments were performed in independent triplicates and two technical replicates were harvested (*n* = 2) from each Schott flask batch culture (*n* = 3), totaling *n* = 6. The data are expressed as mean ± standard deviation (SD) values or represented using standard deviation bars in the graphs from 0 to 192 h of culture (GraphPad software). The Z‐score was chosen as a potential tool for statistical analysis of each metabolite at each time point from 0 to 144 h. The heat map was generated using Prism software (GraphPad), and the clustering analysis was performed using MultiExperiment Viewer (MeV) software [29]. A K‐means clustering (KMC) method was applied to group the metabolic profiles into k clusters using Pearson correlation.

## 3 Results

### 3.1 Kinetics of Cell Growth of *Drosophila* S2, Wild‐ and Recombinant‐Type Strains

**FIGURE 1:**
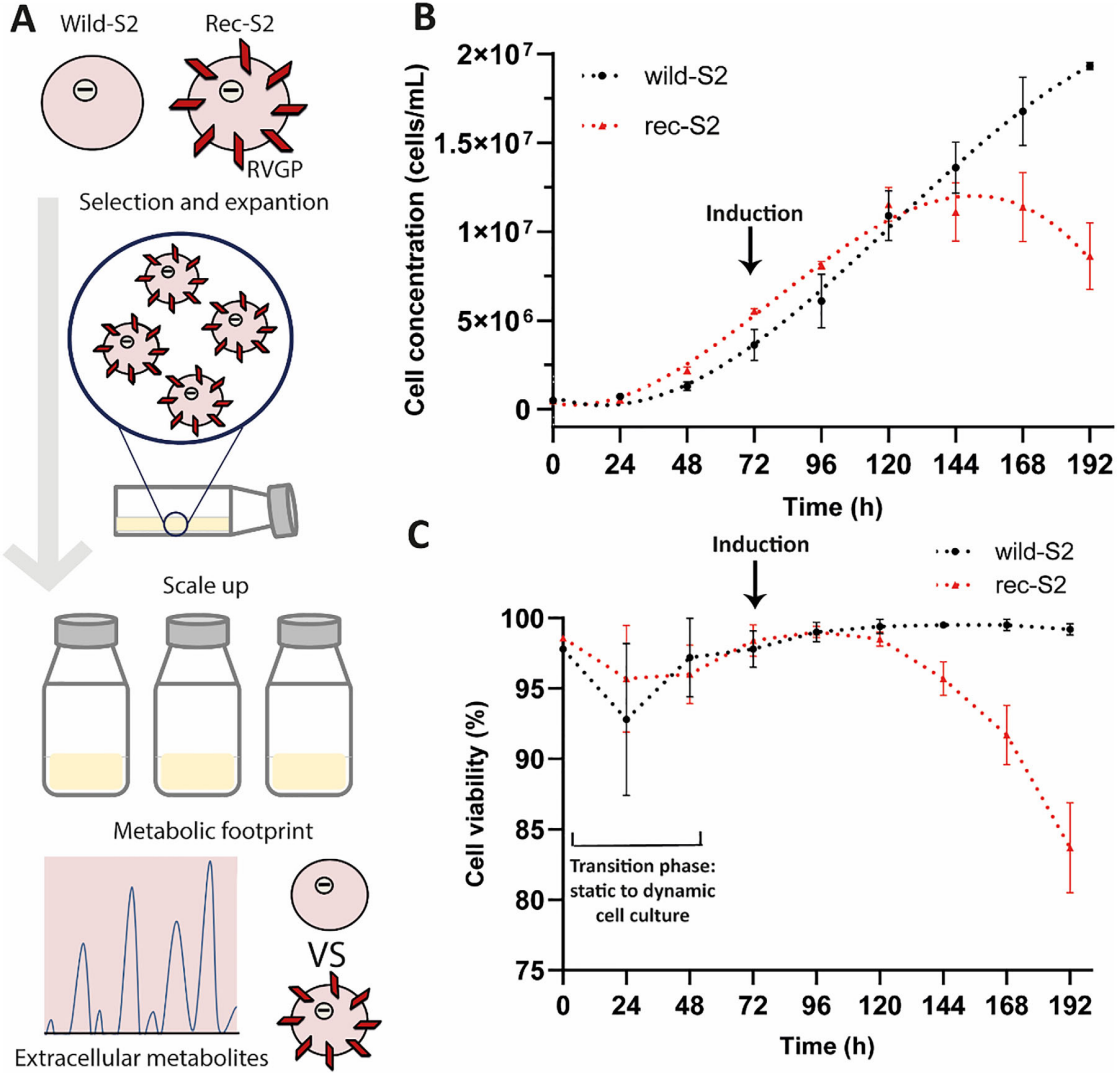
Workflow of the study and evaluation of growth parameters of wild‐type and recombinant S2 cells. (A) Schematic illustration of the workflow for S2 cell culture and metabolism analysis. Comparative metabolic footprint among batch cultures of *Drosophila melanogaster* S2 cells, wild‐type (wild‐S2) and recombinant‐type (rec‐S2) strains. (B) Growth curves (linear scale) of rec‐ and wild‐types and (C) cell viability of rec‐ and wild‐types inoculated with 0.7 mM CuSO_4_ at 72 h of culture (indicated by black arrow) for real or mock RVGP expression. Three independent precultures were used to inoculate three Schott flasks in batch cultures for each strain, with two technical replicates per batch.

A schematic illustration of the bioprocess in this work for producing RVGP, with a focus on the comparative metabolic footprints of the wild‐ and recombinant‐type strains, is presented in Figure 1A. To identify all the phases of the cell culture growth profile using wild‐ and rec‐type strains, cell concentration (Figure 1B) and cell viability (Figure 1C) were assessed at regular intervals of 24 h. In the first 0–24 h, S2 cells were adapted from a semi‐adherent culture to suspension culture. Thus, no cell proliferation was noticed during this time (Figure 1B), and reductions in cell viability from 97.8% to 92.8% for the wild‐type and from 98.6% to 95.7% for the rec‐type were noticed (Figure 1C). After this adaptation period, the exponential phase started at 24 h. Employing a linear correlation to determine the specific growth rates (*µ*) of both cell types, 120 h was identified as the last moment at which the cells could maintain their maximum specific growth rate. The deceleration and death phases were identified for the rec‐type from 120 to 192 h, with a reduction in cell concentration (Figure 1B) from 1.15 × 10^7^ to 8.61 × 10^6^ cells/mL and a decrease in cell viability (Figure 1C) from 98.5% to 83.7%. Interestingly, the same reduction in cell concentration was not observed with the wild‐type, either in the presence or absence of copper sulfate (Figures S1 and 1B,C, respectively). Cell concentration increased from 1.09 × 10^7^ to 1.93 × 10^7^ cells/mL, along with the maintenance of high cell viability (99.2% to 99.4%) in the absence of copper sulfate, even after 192 h in culture (Figure 1B).

To determine cell culture parameters and specific growth rates for both S2 cell lines, the interval between 0 and 120 h was considered. However, since this is an inducible expression system and the addition of CuSO_4_ impacts cell growth rates, this phase was divided into two distinct phases, pre‐ and post‐induction. In the pre‐induction period (µ_before_), corresponding to 0–72 h, a similarity in growth kinetics between both cell lines was observed. The specific growth rates (µ) were 0.029 ± 0.005 and 0.035 ± 0.007 h^−1^ for wild and rec‐S2, respectively. The average time needed for cells to duplicate was 23.92 h and 20.08 h for wild and rec‐S2, respectively. On the other hand, in the post‐induction phase (µ_after_) corresponding to 72–120 h, different growth kinetic profiles were observed for both cell types. The specific growth rates were 0.022 ± 0.003 and 0.014 ± 0.002 h^−1^ for the wild‐type and rec‐S2, respectively. The average duplication time was 31.12 and 48.36 h for the wild and rec‐S2, respectively. When comparing duplication time as a practical parameter to evaluate cellular performance, it increased by 30% from pre‐ to post‐induction in the wild‐type, whereas in the rec‐S2 it showed an increase of 141% over the same period. These data provide evidence that the activation of the inducible promoter to produce the foreign RVGP in the rec‐S2 affects the kinetics of cell growth. All these data are summarized in Table 1.

**TABLE 1:** Cell culture parameters related to growth profile of wild‐type and rec‐S2 cells during RVGP production: Specific growth rate (*µ*); cell culture doubling time (*d*_t_); specific rate of consumption of glucose (q_gluc_); proline (q_pro_); and glutamine (q_gln_); specific production rate of RVGP (q_RVGP_). These parameters were calculated for the pre‐induction period (0–72 h) and the post‐induction period (72–120 h) and were based on the number of viable cells in each batch culture and at each time point.

				Specific rates related to cell growth profile			
				(×10^−11 ^mmol.cell^−1^.h^−1^)			(×10^−6 ^ng.cell^−1^.h^−1^)
Cell type	Time of culture (h)	µ (h^−1^)	*d*_t_ (h)	*q*_gluc_	*q*_pro_	*q*_gln_	*q*_RVGP_
Wild	0 – 72	0.029 ± 0.005	23.92	8.96 ± 4.51	0.79 ± 0.72	1.73 ± 0.49	—
wild	72 – 120	0.022 ± 0.003	31.12	1.90 ± 1.02	0.31 ± 0.14	0.83 ± 0.20	—
rec	0 – 72	0.035 ± 0.007	20.08	11.7 ± 1.69	1.19 ± 0.31	5.14 ± 0.88	—
rec	72 – 120	0.014 ± 0.002	48.36	2.27 ± 0.22	0.30 ± 0.05	0.73 ± 0.07	2.17 ± 0.42

### 3.2 Consumption of Substrates and Production of Metabolites

**FIGURE 2:**
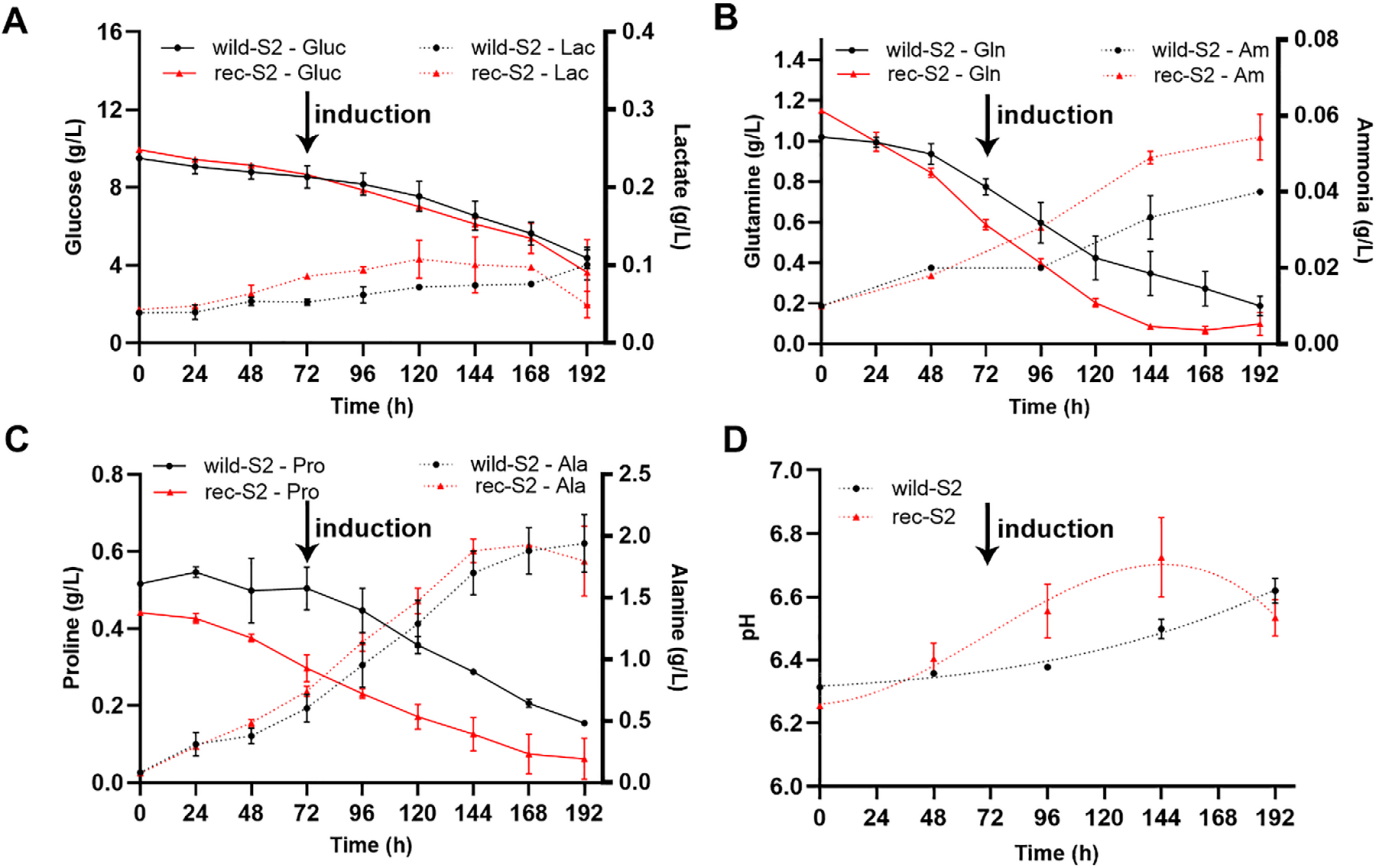
Consumption of the main substrates and production of the main metabolites in batch cultures of S2 cells, wild‐type (wild‐S2) and recombinant‐type (rec‐S2). (A) glucose and lactate; (B) glutamine and ammonia; and (C) proline and alanine. (D) pH variation during 192 h of batch cultures. The induction of the RVGP using CuSO_4_ for the rec‐S2 was performed in a single step at 72 h of the culture (indicated by the black arrows).

During the batch cultures, we identified that the substrates most consumed by both wild‐type and rec‐S2 were glucose, proline, and glutamine. Analyzing Figure 2A–C, it is possible to observe that glucose was not depleted, whereas glutamine and proline in the rec‐S2 reached low values, smaller than 0.2 g/L at 144 h. Glutamine concentration decreased even more significantly after 144 h, likely approaching limiting values. Regarding metabolites, we identified three compounds secreted in significant amounts in both wild‐type and rec‐S2, which were alanine, ammonia, and lactate (Figure 2A–C).

The pH slightly oscillated during the batch cultures from 6.28 ± 0.04 at day 0 (corresponding to the fresh culture medium without cells) to 6.62 ± 0.04 in the wild‐type and 6.53 ± 0.06 in the rec‐type (Figure 2D). This pH fluctuation was favorable, as the optimal pH for S2 cell growth ranges from 6.2 to 6.4 when cultured at the ideal temperature of 28°C [17]. Thus, cell growth was prioritized during the pre‐induction period (0–72 h), and no pH correction was performed in any batch culture. In the post‐induction period (72–120 h), the biosynthesis of RVGP took place, and the recombinant protein accumulated in the slight acid pH although the ideal pH would be as close as possible to the optimum pH of RVGP, that is, 7.2 [30]. A limitation of our experimental model is that the Shott flask system could not maintain pH and oxygen consumption at optimal levels in real time. Nevertheless, this same bioprocess was previously performed by our team using the wave‐induced bioreactor [17] and stirred vessel bioreactor [18], in which cell culture parameters were monitored. In these systems, the pH and oxygen levels followed the same trend as the one observed in small‐scale experiments (Schott flasks). Thus, these process variables did not significantly affect the results. Herein, in the Schott flask batch cultures, the pH was already spontaneously increasing, but a correction to pH 7.2 using buffers was performed when the experiments were concluded. This strategy was adopted to prevent deleterious effects on both RVGP structure and function, since it is a transmembrane protein.

Since the profiles of consumption of glucose, proline, and glutamine, as well as the production of lactate, alanine, and ammonia were similar in both wild‐type and rec‐S2, the specific substrate consumption rates (*q*_s_) were calculated to distinguish their metabolic behaviors by determining consumption values per cell per hour. The consumption of glucose (q_gluc_), proline (q_pro_) and glutamine (q_gln_), as well as the production of RVGP (q_RVGP_) in the pre‐ and post‐induction periods, are shown in Table 1. It is evident that glucose, proline, and glutamine were consumed at much higher rates before the addition of the inducer in both wild‐type and rec‐S2. Interestingly, glucose and proline showed similar trends across both S2 cell types, whereas glutamine showed the opposite trend. Glutamine was consumed 3 times more by the rec‐S2 before RVGP induction than by wild‐type. However, after RVGP induction, the glutamine consumption rate was similar in both cell types. Thus, we aimed to investigate whether the difference in glutamine consumption rates was triggered by the biosynthesis of RVGP in rec‐S2 cells.

### 3.3 Changes in the Amino Acid Profile for the Biosynthesis of RVGP

**FIGURE 3:**
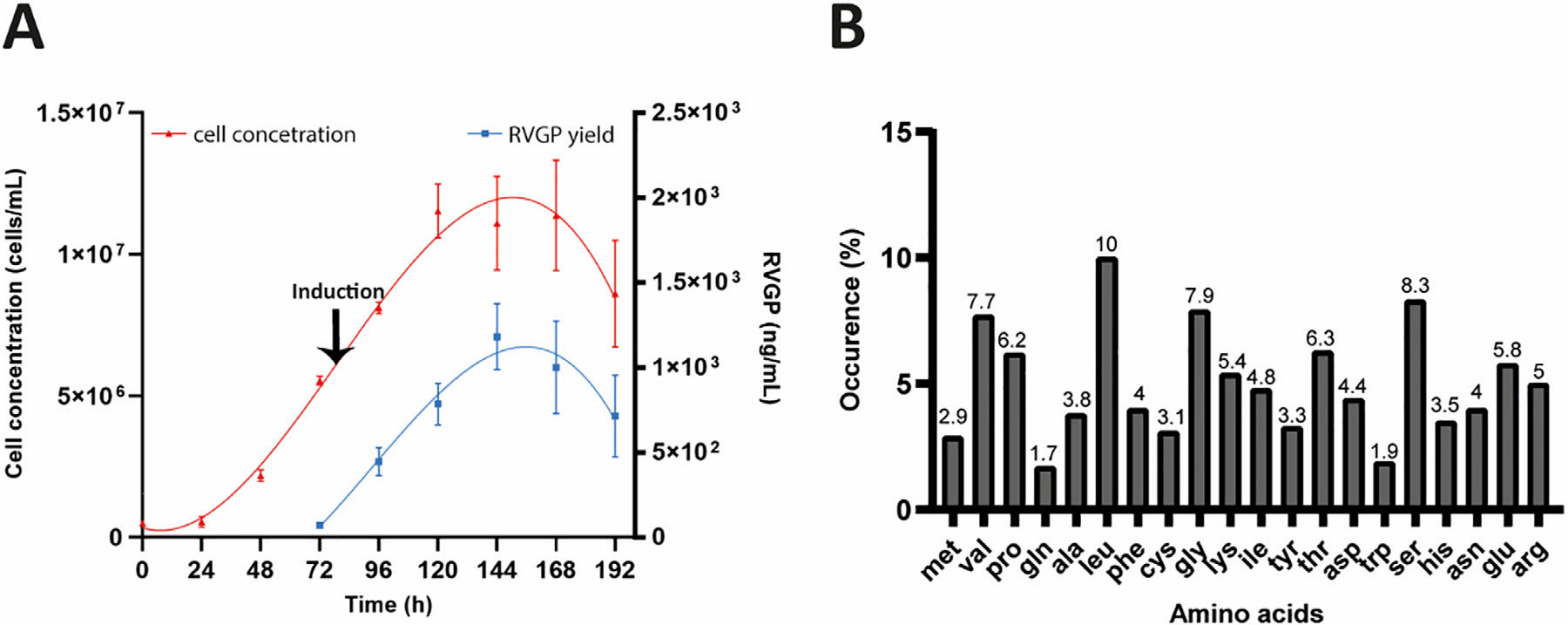
(A) Biosynthesis of the RVGP and growth curve of the recombinant‐type of S2 cells. Three independent batch cultures were performed until 192 h of culture. The induction of the RVGP using CuSO_4_ was performed in a single step at 72 h of the culture (indicated by the black arrow). (B) Amino acid composition of the RVGP, deduced from the genomic sequence of the *Pasteur* rabies virus strain (GenBank accession number M13215.1).

The biosynthesis of the RVGP was analyzed (Figure 3A). During the post‐induction period (72–120 h), high RVGP production was observed, reaching 2.17 × 10^−6^ ng×cell^−1^×h^−1^. After this period, from 144 to 192 h, the specific production rate of RVGP assumes negative values (‐7.14 × 10^−7^ ng×cell^−1^×h^−1^), indicating RVGP degradation. When analyzing the RVGP biosynthesis profile alongside cell viability, 144 h is the maximum time point for concluding the batch cultures and harvesting RVGP while ensuring its quality. At 144 h, a total amount of 1.18 × 10^6 ^ng RVGP per liter of the Shott flask was harvested. Given that approximately 1 × 10^6^ ng/L of RVGP would be sufficient for the immunization tests on 500 mice, 144 h was confirmed to be satisfactory. Hence, this lab‐scale bioprocess demonstrates high potential for pursuing further immunization studies.

Previously to this work, the immunogenicity of the RVGP produced by S2 cells was assessed. Antibody titer levels after mice immunization and survival to rabies virus challenge were reported [17]. The results showed that mice immunized with S2 produced RVGP or with a commercial rabies vaccine have achieved neutralizing antibody levels of 2.92 ± 0.61 and 3.40 ± 1.59 EU/mL, respectively, and successfully survived the challenge with rabies virus [17].

The amino acid composition of the RVGP was deduced from the genomic sequence of the *Pasteur* rabies virus strain (GenBank accession number M13215.1). Twenty amino acids were identified as components of the RVGP (Figure 3B), and the highest occurrence was observed for leucine (10.0%), serine (8.3%), glycine (7.9%), and valine (7.7%). Hence, the uptake of these four amino acids during RVGP biosynthesis (72–120 h) was analyzed to distinguish their uptake profiles per cell per hour. The uptake of leucine (q_leu_), serine (q_ser_), glycine (q_gly_) and valine (q_val_) in the pre‐ and post‐induction periods can be seen in Table 2.

**TABLE 2:** Specific consumption rates of amino acids related to RVGP biosynthesis by wild‐type and rec‐S2 cells during RVGP production: Uptake of leucine (q_leu_); serine (q_ser_); glycine (q_gly_) and valine (q_val_). These parameters were calculated for the post‐induction period (72–120 h) and were based on the number of viable cells in each batch culture and at each time point.

Cell type	Time of culture (h)	Specific uptake rates of amino acids related to RVGP biosynthesis (×10^−11 ^mmol.cell^−1^.h^−1^)			
		*q*_leu_	*q*_ser_	*q*_gly_	*q*_val_
wild	72 – 120	0.22 ± 0.04	0.18 ± 0.08	0.13 ± 0.06	0.26 ± 0.01
rec	72 – 120	0.13 ± 0.03	0.06 ± 0.05	0.08 ± 0.03	0.15 ± 0.04

According to the results in Table 2, during the period of biosynthesis of RVGP (72–120 h), there were differences in the uptake of the four amino acids with the highest occurrence in RVGP composition. The rec‐S2 used 67% less serine, 42% less valine, 41% less leucine, and 38% less glycine than the wild‐type from 72 to 120 h. Hence, it seems that there was less need for the amino acids with the highest abundance in RVGP by the recombinant type. Interestingly, there was no difference between the uptake of these amino acids in both wild‐S2 and rec‐S2 cells in the period before induction (0–72 h). At the same time, no RVGP was induced, thus not produced (Table S1).

### 3.4 Changes in Organic Acid Profiles for the Biosynthesis of RVGP

**FIGURE 4:**
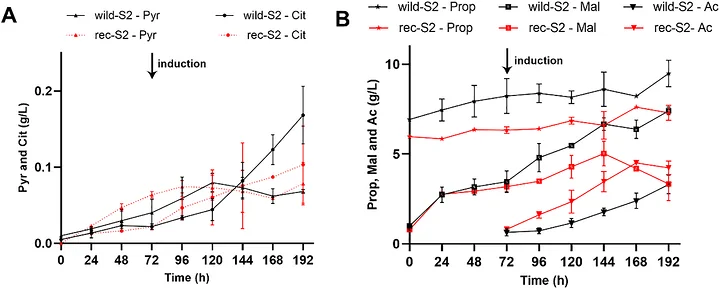
Extracellular concentrations of organic acids of batch cultures of wild‐type and rec‐S2 cells for 192 h. (A) Pyruvate and citrate; (B) Propionate, malate, and acetate. The induction of the RVGP expression using CuSO_4_ in the rec‐type was performed in a single step at 72 h (indicated by black arrows).

The extracellular concentrations of acetate (Ac), citrate (Cit), lactate (Lac), malate (Mal), propionate (Prop), and pyruvate (Pyr) were analyzed during the 192 h (Figure 4). The profiles of pyruvate and citrate were similar in both wild‐ and rec‐ types (Figure 4A), and the identification of these organic acids was observed at lower levels, ranging from 0.005 to 0.2 g/L. The propionate concentrations were also similar throughout the whole period (Figure 4B), although values for the rec‐S2 were slightly lower due to the variation in the fresh culture medium (5.96 g/L for the batch culture of rec‐S2 and 6.92 g/L for the batch culture of wild‐type). Interestingly, malate profiles were similar in both wild‐type and rec‐S2 until the RVGP induction time at 72 h (Figure 4B). After that, the wild‐type produced malate in higher amounts than the rec‐S2. Regarding acetate, its concentration in the culture medium remained below the detection limit until 72 h of induction. Following the same trend as malate, at 72 h, acetate concentrations in both wild‐ and rec‐S2 were similar, but a markedly different profile was observed after RVGP induction, with rec‐S2 producing higher amounts (Figure 4B). These data show that malate and acetate production are correlated with RVGP production.

### 3.5 Metabolic Footprints of Wild‐Type and rec‐S2 cells During 144 h of Batch Cultures

**FIGURE 5:**
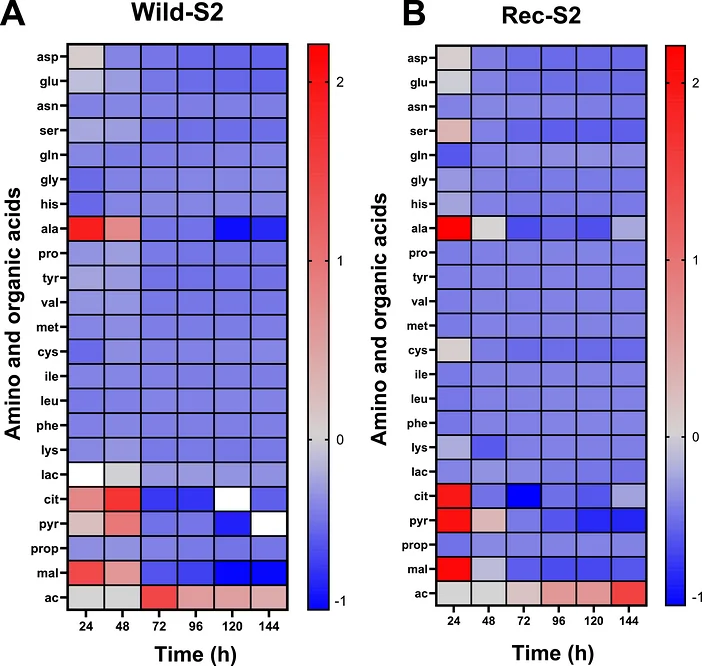
Heat map of wild‐type and rec‐S2 cells. Metabolites were accessed during RVGP production for 144 h in batch cultures, and the heat map was established using the Z‐score (Equation 5). Cold colors (blue) indicate diminished metabolite levels, suggesting consumption, and warm colors (red) indicate compound accumulation. Three independent batch cultures were performed (*N* = 3).

**FIGURE 6:**
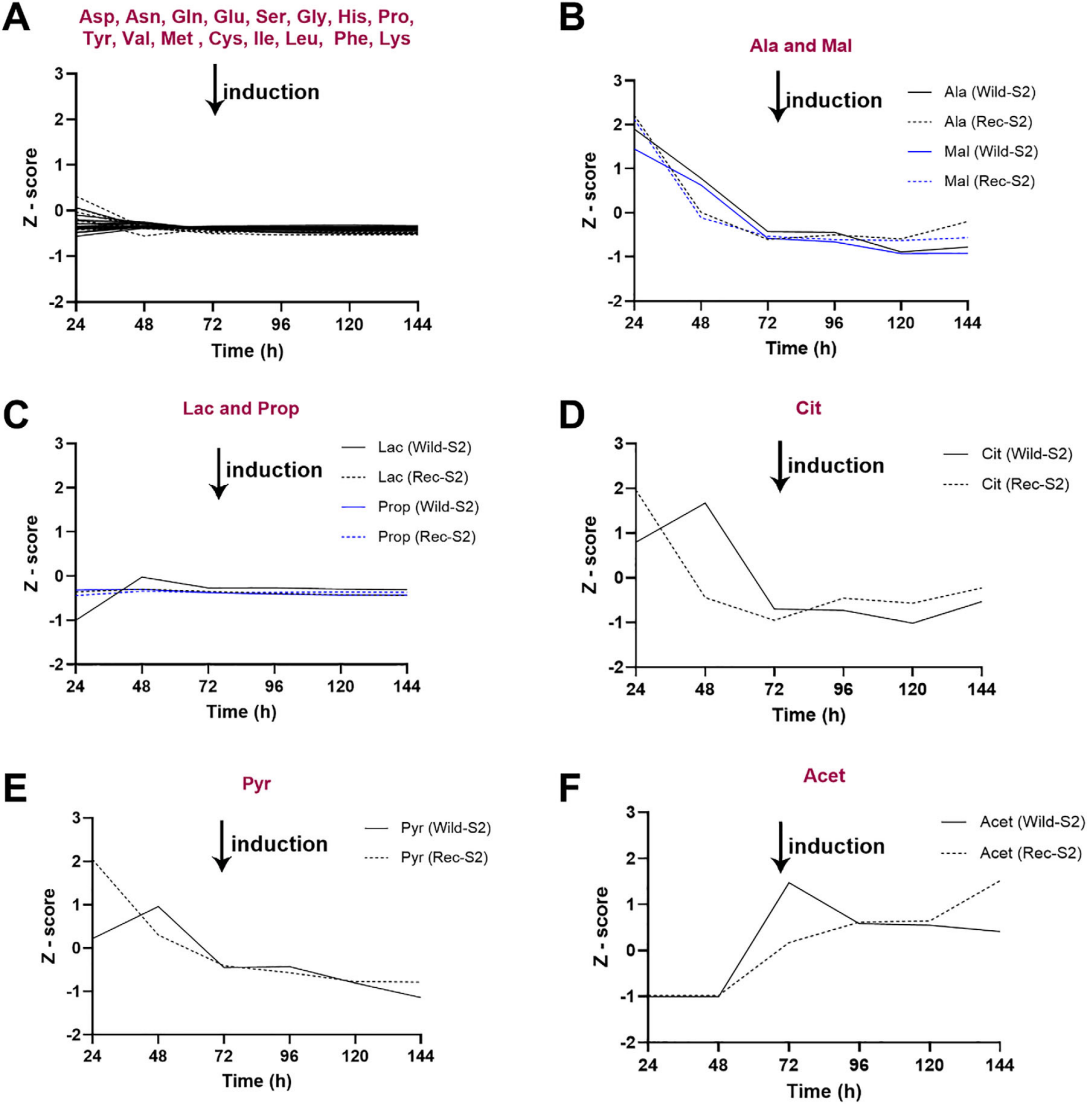
Clustering of the metabolic profiles. To identify associations or similarities of extracellular compounds during RVGP production for 144 h in batch cultures of wild‐type and rec‐S2 cells, metabolic profiles were clustered. K‐Means Clustering (KMC) produced clusters based on the KMC input parameters (50‐iteration upper limit). The Z‐score was obtained by employing Equation 5. The clustering process identified six clusters, which were composed of (A) Asp, Asn, Gln, Glu, Ser, Gly, His, Pro, Tyr, Val, Met, Cys, Ile, Leu, Phe and Lys; (B) Ala and Mal; (C) Lac and Prop; (D) Cit; (E) Pyr and (F) Acet. The induction of the RVGP using CuSO_4_ in the rec‐S2 batch culture was performed in a single step at 72 h (indicated by the black arrows).

To correlate the 23 amino and organic acids (the most relevant ones presented in Figures 2 and 4), the values were normalized to cell concentrations (i.e., all values were divided by the corresponding number of viable cells at each time point), and two analyses were employed. First, a metabolomics footprint (Figure 5) to investigate the repertoire of all consumed or secreted metabolites. Then, the metabolic footprint was clustered to identify associations or similarities between cell types (Figure 6).

Analyzing the heatmap, lower Z scores (including negative values) indicate that the metabolites are diminished, suggesting they were consumed, shown in cold colors (blue, decreasing values). Higher Z scores indicate metabolite elevation, suggesting that more of the compounds have been produced by the cells. Higher Z scores are shown in warm colors as an accumulation of compounds (red, increasing values). The amino acids asparagine, glutamic acid, serine, cysteine, and lysine showed lower consumption rates during the first 24 h in both cell lines, in contrast to glutamine, which showed higher consumption rates in the rec‐S2. As expected, alanine has shown a completely different behavior since it is extensively produced during S2 cell cultures, as mentioned above (Figure 2C). When quantifying compounds found in the fresh culture medium, alanine was found in very small amounts (84.8 mg/L) compared with other amino acids. All other amino acids in cold blue colors indicated consumption by both cell lines. As indicated in Figure 4, organic acids presented different trends in both wild‐type and rec‐S2. While malate and acetate were identified as key players in RVGP production (Figure 4B), when normalizing amino acid concentrations to cell concentrations to obtain the metabolic footprint, citrate, pyruvate, and lactate were also identified as key players, albeit at lower intensity. At 24 h, malate was produced by both cell types in a similar trend, but began to differ from 48 h onwards. At the end of the culture, malate was consumed at a higher rate in wild‐type than rec‐S2. For acetate, both wild‐type and rec‐S2 produced it throughout the culture period, but wild‐type reached a higher production rate at 72 h (before RVGP induction), while rec‐S2 reached it at 144 h. Regarding citrate, wild‐type cells produced it until 48 h, whereas rec‐S2 cells produced it only at some point between measurements 1 and 24 h. Later on, citrate was consumed in both wild‐type and rec‐S2. For pyruvate, both wild‐type and rec‐S2 produced it until 48 h, with rec‐S2 cells producing much higher amounts at 24 h. From 72 h onward, pyruvate was consumed by the cells, or it may have served as an intermediate in another metabolic pathway.

Finally, the heat map was clustered to identify associations or similarities. Six clusters exhibiting different behaviors were observed (Figure 6). The first cluster (Figure 6A) shows that all amino acids analyzed herein followed a close pattern of consumption, except for alanine. In contrast, alanine showed a similar profile to the organic acid malate (Figures 5 and 6B). The third cluster evidenced an association of lactate and propionate (Figure 6C). The fourth (Figure 6D), fifth (Figure 6E), and sixth (Figure 6F) clusters, involving citrate, pyruvate, and acetate, respectively, did not follow a pattern similar to any other metabolite analyzed. Moreover, although they maintained a similar profile between wild‐type and rec‐S2, variances across time points in the cell culture for each cell type were identified.

## 4 DISCUSSION

The production of heterologous proteins often represents a metabolic burden for the host cell due to limited energy and resources to support both cell growth and recombinant protein production [27, 31, 32]. The host cell can often adjust its metabolic fluxes to maintain cell viability while supporting the additional metabolic load. Nevertheless, it is not clear whether the same can be done to maintain heterologous protein production. As a result, cell productivity often fails to meet the required levels for heterologous product production, thereby impairing scale‐up for industrial production. To increase cell productivity, metabolic correlations that may have direct implications for media optimization and yield maximization are crucial. This work revealed several ones and aimed to improve S2 performance for large‐scale upscaling.

Our data have shown a clear impact on the cell growth kinetics of rec‐S2 cells resulting from activation of the inducible promoter for RVGP production. A substantial reduction in cell concentration (1.15 × 10^7^ to 8.61 × 10^6^ cells/mL) and cell viability (98.5% to 83.7%) was seen, while in the wild‐type, the number of cells almost doubled (1.09 × 10^7^ to 1.93 × 10^7^ cells/mL) with the maintenance of high cell viability of around 99% even at 192 h. As previously described [17], once the metal‐inducible promoter is activated to produce the foreign protein, there is a period for the production to take place, as well as for the subsequent incorporation of the RVGP into the cell membrane. This is certainly the main reason behind the similar growth kinetics of wild‐type and rec‐S2 cells until 96 h (24 h after induction). Afterward, we hypothesize that cellular resources were redirected toward glycoprotein production, thereby affecting cell growth rate and viability. This trend has been observed in previous studies and is often referred to as a metabolic shift [17, 32, 33]. If there were no limiting substrate, space, or inhibition factor, cells would maintain their exponential growth. Thus, we aimed to determine whether this noticeable metabolic shift was related to insufficient substrates or the presence of inhibitory factors. Herein, we revealed glutamine as a substrate limitation for rec‐S2 cell growth, changes in amino acid routes due to RVGP biosynthesis (leucine, serine, glycine, and valine), and the production of compounds that might inhibit the growth of this cell strain (acetate, pyruvate, citrate, and malate).

Regarding substrates for cell growth, among the 27 compounds quantified herein, the most consumed were glucose, glutamine, and proline for both wild‐type and rec‐S2. In a previous study with another S2 cell line, glucose, glutamine, asparagine, serine, and proline were the main nutrients consumed [34]. Hence, a variety of amino acids can be used. Additionally, it is well known that *Drosophila sp*. can use various carbohydrate sources for energetic pathways, such as glucose, fructose, maltose, sucrose, galactose, xylose and lactose, emphasizing the high metabolic flexibility of S2 cells considering substrate uptake [35].

Often, glucose is the most employed one, and it is very abundant in the culture medium used in this study (9.4 g/L). As expected, glucose followed a similar trend in both wild‐type and rec‐S2 and was not depleted in this bioprocess. Thus, it was not the cause of the growth limitation of the rec‐S2 cells. Glutamine is also known to provide energy and plays a key role as a precursor for biomass synthesis in S2 cell cultures [5, 14]. Herein, we found that glutamine was consumed 3 times more by the rec‐S2 cells before RVGP induction (Table 1), reaching constant values close to zero at the final time points (144–192 h) of the culture (Figure 2B). This higher glutamine uptake correlates with a 2.5‐fold increase in cell growth rate in the same period. Analyzing the growth curve of rec‐S2 (Figure 1A), a clear transition to the stationary and death phases was also noticed from 144 to 192 h. Thus, glutamine was a limiting substrate for rec‐S2 growth. Indeed, preliminary studies have shown that a lack of either glucose or glutamine generally results in cell growth limitation [14]. Surprisingly, the amount of glutamine was reduced by approximately half when comparing the amount in the commercially available Sf‐900 III culture medium analyzed herein (1.2 g/L) with the previous commercially available Sf‐900 II concentration (more than 2 g/L [20]). The Sf‐900 culture medium can be used for insect cells in general, but it was formulated specifically for *Spodoptera frugiperda (Sf9)* and glutamine is not an essential amino acid for Sf9 cells, while it is for *Drosophila melanogaster* and even more for recombinant *D. melanogaster* strains [36]. Thus, glutamine supplementation is needed to produce RVGP using Sf‐900 III at high cell concentrations. In fact, the culture medium could have been supplemented at the start of the culture period (0 h) or at least after 72 h, when glutamine begins to approach limiting levels (∼0.5 g/L). The supplementation at the beginning of the culture period should not cause any severe consequences to the accumulation of glutamine metabolites, since higher amounts had already been used in the previous formulation of the commercial Sf‐900 II culture medium. In principle, this adjustment could also be considered when producing other proteins in S2 cells, but additional testing would be required to determine the optimal conditions. Accordingly, a previous study also showed that glutamine was depleted when using supplemented IPL‐41 medium [34]. Finally, proline plays a different role in insect cells than in mammalian cells, and it is considered another essential energy source for S2 cells [5, 14, 37]. The addition of 8.7 mmol/L proline to the Sf‐900 II medium nearly doubled the specific cell growth rate compared with a proline‐free culture medium [14]. Herein, proline was neither depleted nor supplemented. Thus, it also seems not to have been the cause of cell growth limitation in rec‐S2 cells.

Regarding substrates for RVGP biosynthesis, we found that leucine, serine, glycine, and valine are the most abundant amino acids in RVGP composition. Interestingly, serine is a known energy source for insect cells, and it can be converted to glycine. A previous report demonstrated that glycine is of utmost importance for cell growth for *Drosophila* sp. [38]. Glycine and valine are both often consumed during exponential insect cell growth and are also used for non‐foreign protein biosynthesis [35, 39]. The four amino acids required for RVGP biosynthesis (leucine, serine, glycine and valine) were consumed by rec‐S2 cells in much lower amounts during the period of RVGP biosynthesis when compared to wild‐type. This reveals that the rec‐S2 may orient the consumption of these amino acids toward RVGP biosynthesis, thereby reducing cell growth, whereas the wild‐type would use these amino acids solely for cell growth. This indicates two potential metabolic routes operating in this bioprocess for RVGP biosynthesis using the rec‐S2.

Regarding the production of compounds that might inhibit rec‐S2 cell growth, several require proper attention. Alanine was produced in high amounts and was the only amino acid for which no association was observed in the metabolic footprint clustering analysis performed. Excessive amounts of alanine produced by insect cells have been reported and are often associated with partial oxidation of glutamine via the alanine aminotransferase system [14, 17]. Ammonia was also produced in higher amounts, and it is associated with alanine excess, since alanine production is described as an efficient strategy to reduce ammonia accumulation [40, 41]. This can be considered a clever metabolic mechanism, since high levels of ammonia might affect protein quality [42], and the distribution of glycan structures [43], thus compromising the proper glycosylation of RVGP. Alanine levels can also depend on other substrate pathways since alanine consumption was observed during proline and glucose exhaustion [14, 36]. Herein, proline and glucose were not limiting substrates, but glutamine was. Thus, the production of alanine at the beginning of both S2 cultures, followed by a consumption trend at the end of the cultures, might be related to low glutamine levels in wild‐type and its exhaustion in rec‐S2 at the end of the culture period. Hence, the excess of alanine seemed not to be harmful, and this amino acid is even considered an energy source to form glycogen [44].

Organic acids were produced in different trends in wild‐type and rec‐S2. When comparing their levels to cell concentrations in the metabolic footprint analysis (Figure 5), lactate, acetate, pyruvate, citrate, and malate were considered as key players. Consistent with other studies, lactate was produced by S2 cells via glucose metabolism, but at much smaller amounts than in mammalian cells [6]. This amount would not be enough to cause growth inhibition or affect RVGP quality. Regarding acetate, recombinant protein production may be triggered by excess sugar, a phenomenon already observed in other biological platforms, such as *Escherichia coli* and *Saccharomyces cerevisiae*, leading to acetate production under aerobic conditions [45, 46, 47]. This path starts with glucose being converted to pyruvate midway through the route, with acetate as the final by‐product. Acetate is a detrimental by‐product that can reduce carbon efficiency, increase cell maintenance, and inhibit cell growth [45, 46]. This route appears to be an inefficient means of energy generation, and although it has been described primarily in microorganisms (e.g., *E. coli and S. cerevisiae*), we hypothesize that carbon overflow from excess glucose might also trigger the accumulation of acetate in *Drosophila sp*. as well. Following this pathway, it is clear that excess pyruvate is produced, since it acts as an intermediate in acetate production [45, 46]. Hence, acetate and pyruvate may be inhibiting growth in the rec‐S2 cells. Moreover, this acidic stress was amplified by RVGP production, as the rec‐S2 cells exhibited much higher acetate levels at the end of the cell culture than wild‐type cells. Herein, chemical strategies for controlling pH can also be considered to prevent an acidic environment or to convert acetate and pyruvate into less toxic forms. Finally, citrate and malate are intermediates of the Krebs Cycle that release energy derived from carbohydrates. Both compounds were previously observed during recombinant protein production in microorganisms such as *E. coli* and *S. cerevisiae* [47, 48]. To the best of our knowledge, this is the first report of citrate and malate production in S2 insect cell culture.

Lastly, we analyzed whether the presence of CuSO_4_ used for RVGP induction could inhibit S2 cell growth due to heavy‐metal‐related toxicity or by influencing on other cellular pathways. To this extent, experiments were also performed with wild‐type cells in the presence of CuSO_4_ (Figure S1). As expected, no cytotoxic or inhibitory effects were observed at the concentration levels analyzed.

During this study, we also identified a new compound present at high concentration in Sf900‐III culture medium that was highly consumed by the S2 cells, but could not be precisely identified. Despite several attempts, including selective chromatographic isolation of this compound, confirmation of its high purity, lyophilization, and mass spectrometry analysis, it was not possible to precisely determine its composition. However, several characteristics of this compound could be identified. It showed an unusually dark blue, oily appearance after lyophilization, which we suspected was due to copper complex formation (Figure S2). Also, the molar mass of around 158 g/mol, absence of benzene rings, ketone, and methyl groups, and presence of seven or eight carbons, including two carbonyl groups, were identified as its main characteristics. Furthermore, a clear instability was observed, as it rapidly produced smaller compounds during mass spectrometry analysis. Table S2 presents over 30 compounds that we initially suspected might be the one isolated in our work. However, mass spectrometry testing revealed that none of them fully matched the compound in question. In any case, we believe this evidence might help other research groups investigating insect cell lines.

S2 cells are very robust and grow rapidly in serum‐free medium either as loose monolayers or in suspension without microcarriers. S2 cultures in bioreactors can easily reach densities of up to 70 million cells/mL, orders of magnitude higher than other insect cell lines [6]. Most recently, by assessing various bioprocess intensification strategies using S2 cells, maximum viable cell concentrations of 31.6, 69.5, and 210 million cells/mL were reported for batch, fed‐batch, and perfusion modes, respectively [49]. Furthermore, S2 cells do not require a precisely controlled CO_2_ atmosphere or temperatures as high as those required by mammalian cells, since they grow very well at 28°C and can achieve superior glycosylation compared with other insects or microorganisms [17]. These characteristics are of great value in using S2 cells as platforms for industrial‐level protein expression of complex proteins. Despite these advantages, only a few reports have analyzed S2 cell metabolism in a more complete repertoire as performed here. Hence, proposing metabolic pathways and analyzing the role of each nutrient in cellular metabolism remain challenging.

Our work provides data on cell growth, metabolic rates, and metabolomic analysis using Z‐score and clustering tools of 27 compounds quantified over 192 h of batch cultures. Using this metabolic footprint, changes in rec‐type cells at a global level during recombinant protein production were revealed. Our results suggest an impact on the growth kinetics of rec‐S2 cells after activation of the copper‐inducible promoter that controls RVGP expression. We identified one substrate limitation (glutamine), several changes in amino acid routes due to RVGP biosynthesis (leucine, serine, glycine, and valine), and several metabolites that potentially affected the growth of rec‐S2 cells (acetate, pyruvate, citrate and malate). Hence, our work revealed metabolic correlations in S2 cells that may have direct implications for media optimization and yield maximization, thereby improving S2 performance for scale‐up.

## Author Contributions

**Monize Caiado Decarli**: conceptualization, methodology, investigation, data curation, writing – original draft, and writing – review & editing. **Diogo Peres dos Santos**: validation and formal analysis. **Daniela Matilde Correia**: software, formal analysis and data curation. **Amadeus Gomes de Azevedo**: investigation and data curation. **Claudio Alberto Torres Suazo**: conceptualization, resources, supervision, and funding acquisition. **Ângela Maria Moraes**: conceptualization, writing – review & editing. **Renato Mancini Astray**: conceptualization, resources, writing – review & editing, supervision, project administration, and funding acquisition. All authors read and approved the manuscript.

## Funding

The authors would like to acknowledge the support for this research by the CNPq (National Council for Scientific and Technological Development, Brazil), grant numbers 402439/2013‐9 and 314724/2021‐4. Monize Caiado Decarli acknowledges the Coordination for the Improvement of Higher Education Personnel (CAPES/Brazil) for her fellowship and the University Medical Center Groningen funds (Groningen, The Netherlands). Daniela Matilde Correia acknowledges CAPES (Atração de Jovens Talentos—Process 064922/2014‐01).

## Ethics Statement

This declaration is not applicable.

## Conflicts of Interest

The authors declare no conflicts of interest.

## Supporting information




**Supporting File**: biot70307‐sup‐0001‐SuppMat.docx.

## Data Availability

The data that support the findings of this study are available from the corresponding author upon reasonable request.
