# Supplementary material for: Metabolic Footprint of Drosophila S2 Cells: Findings During the Production of a Recombinant Rabies Virus Glycoprotein
Source: Biotechnol J. 2026 Sep 10;21(9):e70307. doi: 10.1002/biot.70307 (PMC13562972; doi:10.1002/biot.70307)
Supplement: Supplementary file 1 [file BIOT-21-e70307-s001.docx]

**Metabolic Footprint of Drosophila S2 Cells: Findings during the Production of a Recombinant Rabies Virus Glycoprotein**

**Authors:** Monize Caiado Decarli^a,c,d,*^, Diogo Peres dos Santos^a,e^, Daniela Matilde Correia^a^, Amadeus Gomes de Azevedo^a^, Claudio Alberto Torres Suazo^a,α^, Ângela Maria Moraes^c^, Renato Mancini Astray^b^

**Affiliations:**

^a^ Department of Chemical Engineering, Federal University of São Carlos, Rod. Washington Luís, 3565-905 São Carlos, SP, Brazil.

^b^ Multipurpose Laboratory, Butantan Institute, Av. Vital Brasil 1500, 05503-900, São Paulo, SP, Brazil.

^c^ Department of Engineering of Materials and of Bioprocesses, School of Chemical Engineering, University of Campinas, Av. Albert Einstein, 500, 13083-852, Cidade Universitária “Zeferino Vaz”, Campinas, SP, Brazil.

^d^ Department of Biomaterials & Biomedical Technology, University Medical Center Groningen/University of Groningen, A. Deusinglaan 1, AV 9713, Groningen, The Netherlands.

^e^ National Institute for Bioprocessing Research and Training (NIBRT), A94 X099, Dublin, Ireland

^*^**Corresponding author:** Dr. Monize Caiado Decarli

Contact details: [m.caiado.decarli@umcg.nl](mailto:m.caiado.decarli@umcg.nl)

Permanent address: Department of Biomaterials & Biomedical Technology, University Medical Center Groningen/University of Groningen, A. Deusinglaan 1, AV 9713, Groningen, The Netherlands.

**Supplementary Figures**

**Figure S1:** Batch cultures performed with *Drosophila melanogaster* S2 cells, wild- and recombinant-types. For the wild-type, a secondary batch containing 0.7 mM Cu2SO4 was performed to exclude the possible toxic effect of copper on the induction process.

**Figure S2: A)** Visual aspect of an unusual dark blue oil observed after lyophilization of an unknown compound identified in high concentration in Sf900-III culture medium. **B)** Screenshot of the HPLC while the unknown compound was being selectively harvested, around 11~12 minutes within the registration of more than 2,00 absorbance units since it reached the maximum value of the equipment. A flow rate of 1 mL/min and a UV W486 detector at 210 nm. More details can be found in the materials and methods session.

**Supplementary Tables**

**Table S1**: Specific rates related to RVGP biosynthesis of the wild-S2 cells and recombinant S2 cells during rabies glycoprotein production: uptake of leucine (q_leu_); serine (q_ser_); glycine (q_gly_) and valine (q_val_). These parameters were calculated for the pre-induction period (0 - 72 h) and were based on the number of viable cells on each batch and time point. Negative values can be interpreted as degradation or not enough uptake.

| Cell strain | Time of culture  (h) | Specific rates of amino acids related to RVGP biosynthesis | | | |
| --- | --- | --- | --- | --- | --- |
|  |  | **(×10^-11^mmol.cell^-1^.h^-1^)** | | | |
|  |  | **q_leu_** | **q_ser_** | **q_gly_** | **q_val_** |
| wild-S2 | 0 - 72 | 0.52  ±  0.13 | -0.75  ±  0.24 | 0.79  ±  0.21 | 0.62  ±  0.02 |
| rec-S2 | 0 - 72 | 0.56  ±  0.14 | -1.37  ±  0.45 | -0.17  ±  0.15 | 0.51  ±  0.04 |

**Table S2:** List of compounds that were tested using mass spectrometry, aiming to identify the unknown compound. None of these fully matched the described chemical characteristics.

| **Amino acids** | **Organic Acids** | **Others** |
| --- | --- | --- |
| aspartic acid | succinic acid | N-acetyl-glucosamin |
| glutamic acid | lactic acid | N-acetil-galactosamine |
| asparagine | formic acid |  |
| serine | citric acid |  |
| glutamine | pyruvic acid |  |
| glycine | propionic acid |  |
| histidine | malic acid |  |
| arginine | acetic acid |  |
| threonine | galacturonic acid |  |
| alanine | glucuronic acid |  |
| proline |  |  |
| tyrosine |  |  |
| valine |  |  |
| methionine |  |  |
| cysteine |  |  |
| isoleucine |  |  |
| leucine |  |  |
| phenylalanine |  |  |
| lysine |  |  |
